# Supplementary material for: JAFFAL: detecting fusion genes with long-read transcriptome sequencing
Source: Genome Biol. 2022 Jan 6;23:10. doi: 10.1186/s13059-021-02588-5 (PMC8739696; doi:10.1186/s13059-021-02588-5)
Supplement: Supplementary file 1 — Additional file 1: Fig. S1-8, Table S2-3,7,9. Supporting figures and tables. [file 13059_2021_2588_MOESM1_ESM.pdf]

Supplementary Material for:

JAFFAL: detecting fusion genes with long read transcriptome sequencing

Nadia M. Davidson[1,2,3†], Ying Chen[4], Teresa Sadras[1,5], Georgina L. Ryland[1,5,6], Piers Blombery[1,5], Paul G. Ekert[1,5,7,8,9], Jonathan Göke[4,10], Alicia Oshlack[1,2,5†]

[1]Peter MacCallum Cancer Centre, Victoria, Australia

[2]School of BioSciences, University of Melbourne, Victoria, Australia

[3]The Walter and Eliza Hall Institute, Victoria, Australia

[4]Genome Institute of Singapore, Singapore, Singapore

[5]Sir Peter MacCallum Department of Oncology, University of Melbourne, Victoria, Australia

[6]Centre for Cancer Research, University of Melbourne, Victoria, Australia

[7]Children's Cancer Institute, Lowy Cancer Centre, UNSW, NSW, Australia

[8]School of Women's and Children's Health, UNSW. NSW, Australia

[9]Murdoch Children's Research Institute, Victoria, Australia

[10]National Cancer Centre Singapore, Singapore, Singapore

[†]Corresponding authors: davidson.n@wehi.edu.au and alicia.oshlack@petermac.org

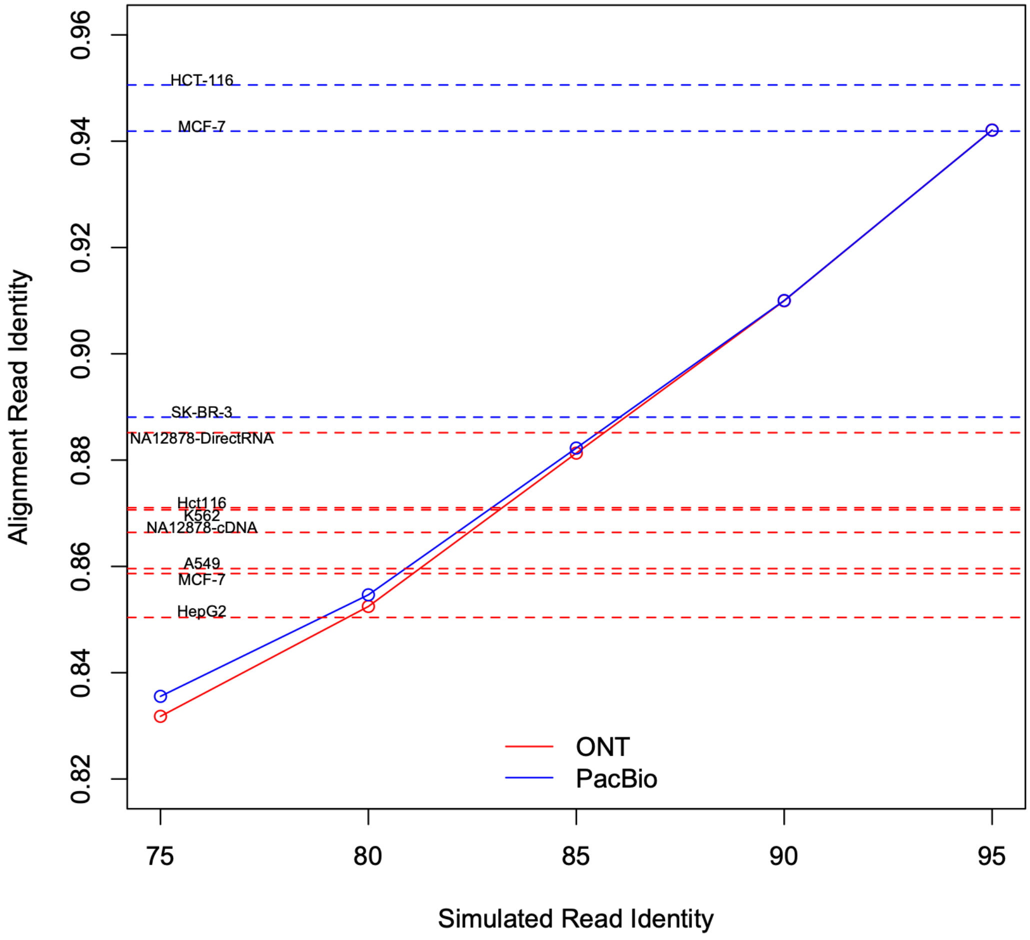

Fig. S1: Simulated and aligned read identifies for the datasets used to evaluate JAFFAL's performance. Solid curves show the simulated read identities for the artificially generated data as provided to badread and their corresponding read identities as measured from alignment to the reference transcriptome with minimap2. The sequence identity is defined by matched bases divided by alignment length. The alignment read identity is generally higher than that simulated because successful alignment is biased towards low error sequence. The horizontal dashed levels correspond to the alignment read identities measured in the real cell line datasets described in the manuscript.

		cRNA - Raw	cDNA - PoreChop
Total Reads Processed		25,418,307	25,286,945
Fusion genes called by JAFFAL	High Confidence	**8**	**9**
	Low Confidence	94	100
	Potential Trans-splicing	412	410

Table S2: The number of fusions called in the non-cancer cell line NA12878 from ONT amplified cDNA before and after processing the data with PoreChop. Two full transcripts including adapters may be sequenced in succession in a single ONT read. To examine the impact of this type of chimera on fusion calling, we applied PoreChop (https://github.com/rrwick/Porechop) to data from NA12878. PoreChop searches and removes adapter sequences and splits reads where internal adapters are found. Approximately 30,000 reads were split by PoreChop. The number of fusions called by JAFFAL remained similar after running PoreChop.

		Direct RNA	cDNA downsamples
Total Reads Processed		14,971,421	14,971,421
Fusion genes called by JAFFAL	High Confidence	**4**	**7**
	Low Confidence	5	43
	Potential Trans-splicing	344	249

Table S3: The number of fusion genes called in NA12878 from ONT direct and amplified cDNA downsampled to the same number of reads. The cDNA sample retains significantly more low confidence fusion calls than the direct RNA sequence even after downsampling to the same number of reads.

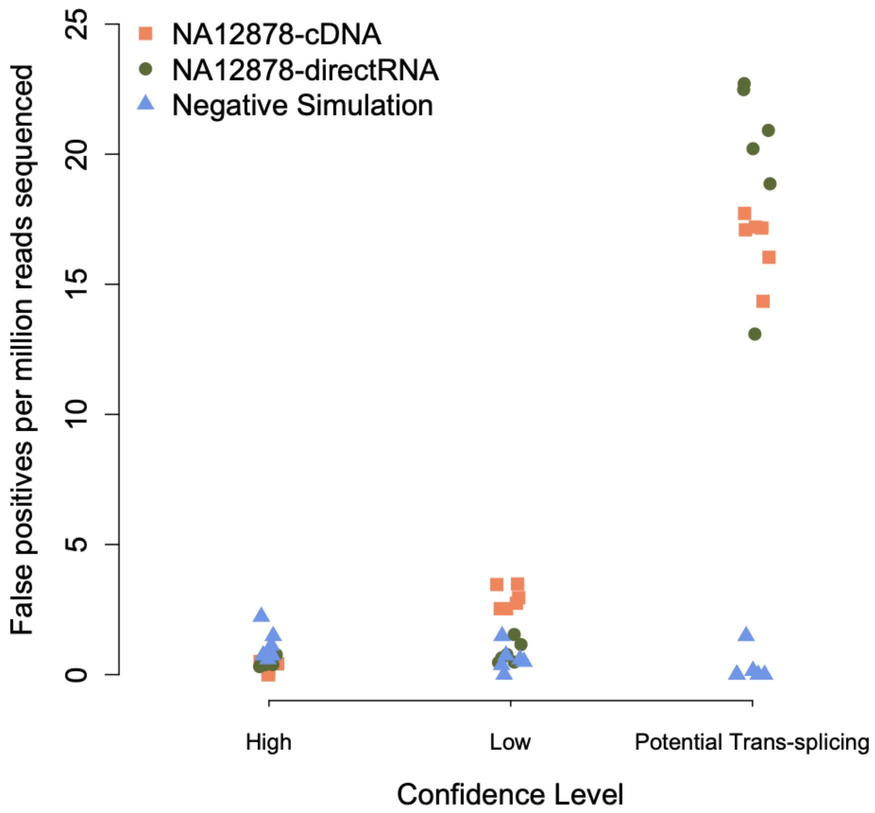

Fig. S2: The number of false positives called at different confidence levels by JAFFAL per million reads sequencing. False positives were counted across three datasets where fusions should not be present: a negative simulation of only non-fused transcripts, amplified cDNA sequencing of the NA12878 non-tumour cell line and direct RNA sequencing of the same cell line. Each dataset was downsampled to 1, 2, 4, 6, 8 and 10 Gbp depth and fusions called with JAFFAL. The number of fusions called per million reads (y-axis) was calculated for each sampling depth and each confidence level of JAFFAL (x-axis). Few fusions were found across any confidence level for the simulation (blue). A high number of false positives consistent with trans-splicing were seen in the direct RNA and cDNA sequencing data (peach and green). A moderate number of false positives consistent with library preparation artifacts were seen in the low confidence cDNA data (peach).

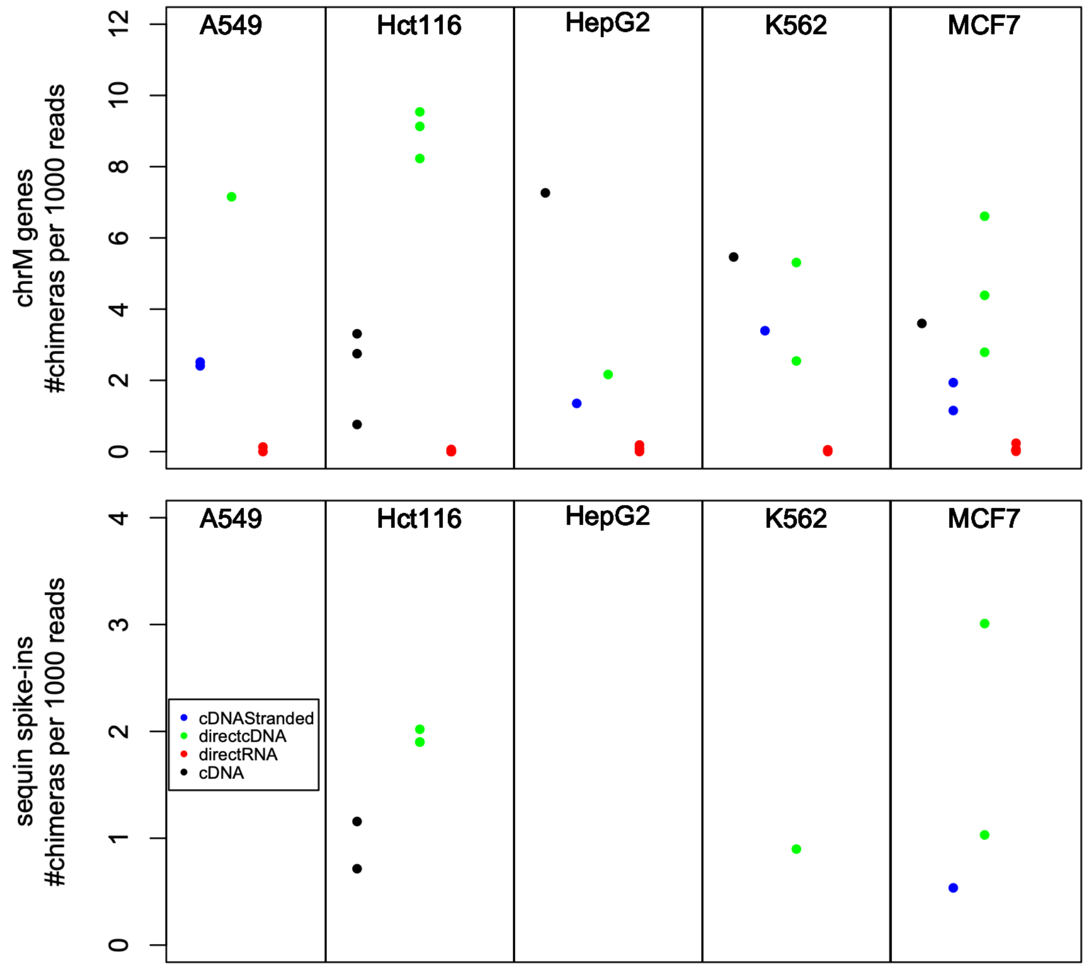

Fig S3: Estimated rates of chimeric artifacts in the SGNex dataset. SGNex data consists of multiple replicates sequenced using different ONT protocols: direct RNA, direct cDNA and amplified cDNA for five cell lines. Two types of known false positives were used as indicators of chimeric artifacts, (top) fusions between a gene on the mitochondrial chromosome and a gene not on the mitochondrial chromosome, and (bottom) fusions between Sequin spike-in transcripts and regular genomic genes, in samples where Sequin spike-ins were added. JAFFAL's default filtering of mitochondrial genes was switched off and its reference supplemented with Sequin sequences. JAFFAL was run on each replicate, and initial candidate fusion reads (ie. those identified after reference transcriptome alignment but before reference genome alignment), were used to calculate the number of chimeric reads. This was then normalised by the total number of reads aligning to either mitochondrial (top) or Sequin (bottom) genes.

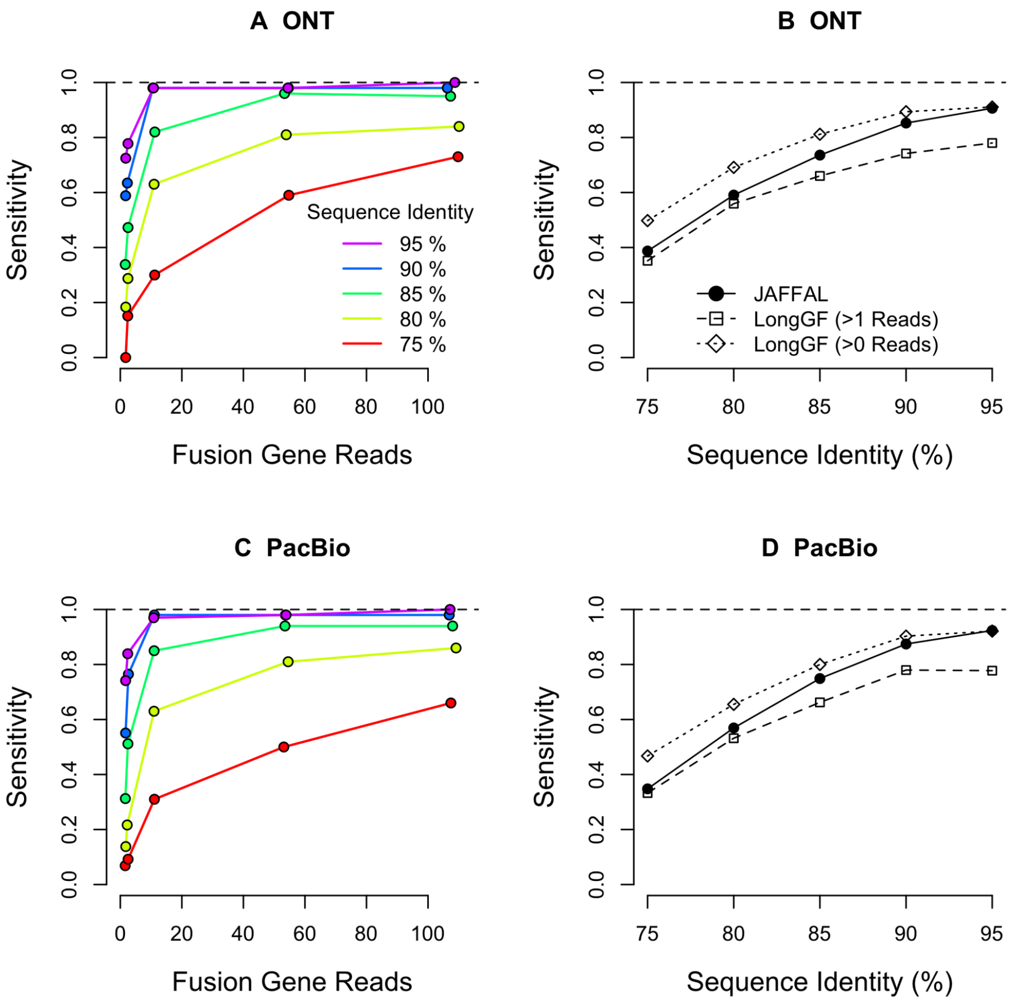

Fig. S4: Fusion finding sensitivity on ONT and PacBio simulated data without background. A) and C) The fraction of simulated fusions detected (y-axis) by JAFFAL across a range of fusion coverage levels (x-axis) and read identity levels (red-purple). B) and D) The fraction of simulated fusions detected (y-axis) by JAFFAL and LongGF for sequence identity levels of 75-95%. ONT and PacBio data were simulated using the same fusion sequence, coverage level and read identity parameters. JAFFAL detected more fusions than LongGF when LongGF was run with default parameters (>1 read support), but fewer when LongGF was allowed to report fusions with just one read support. This intermediate behaviour of JAFFAL is consistent with its reporting fusions with one read support conditional on the breakpoint coinciding with exon boundaries. At low read identities, this condition is more likely to fail due to poor alignment. LongGF reported fewer simulated fusions when background reads were included in the data (Manuscript Figure 2B).

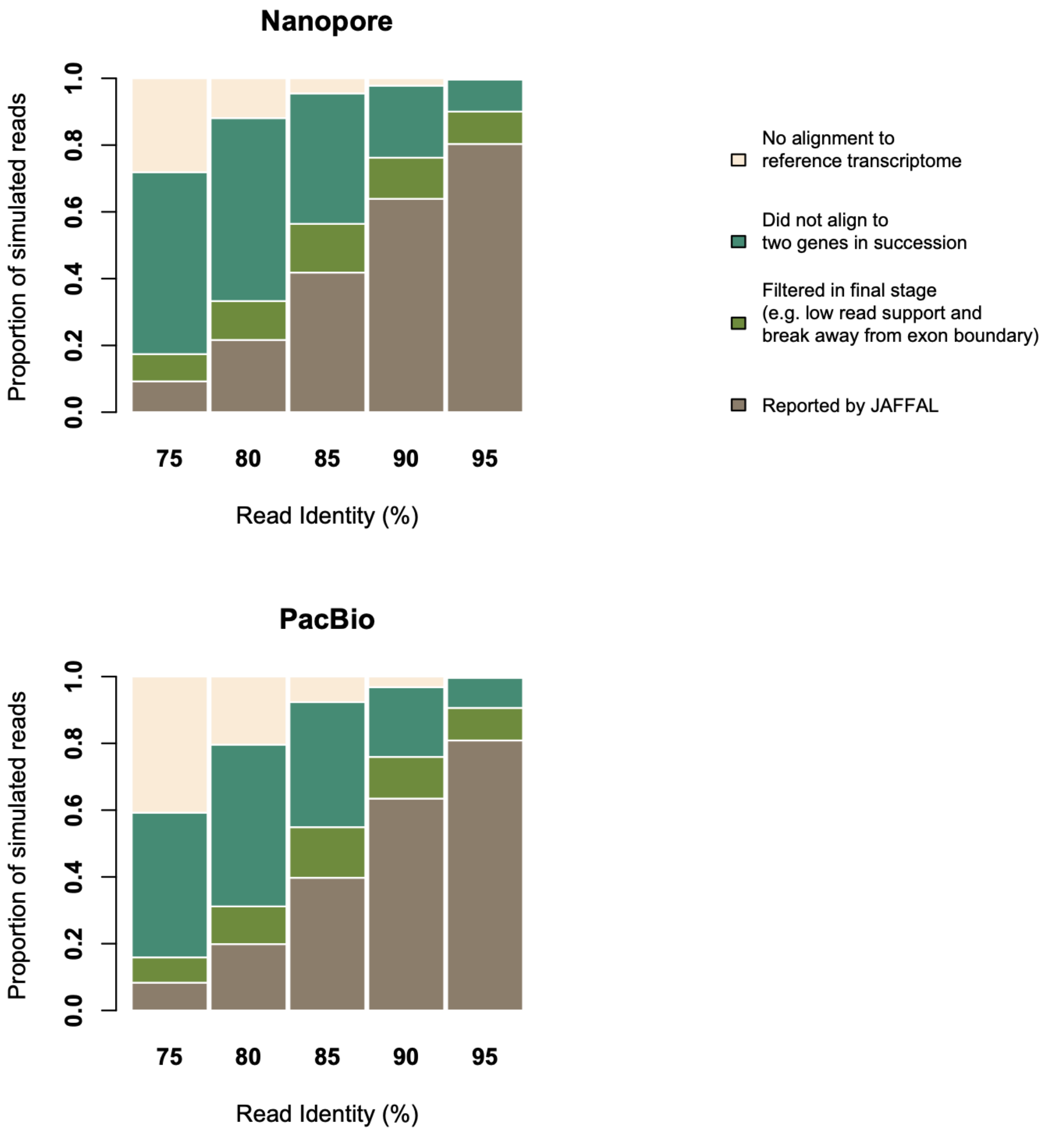

Fig. S5: Proportion of simulated fusion reads lost in various stages of the JAFFAL pipeline. Fusion reads are predominantly lost due to failure to align to the reference transcriptome (cream and dark green). As read identity increases alignment becomes more accurate and a greater proportion of reads are identified by JAFFAL (brown).

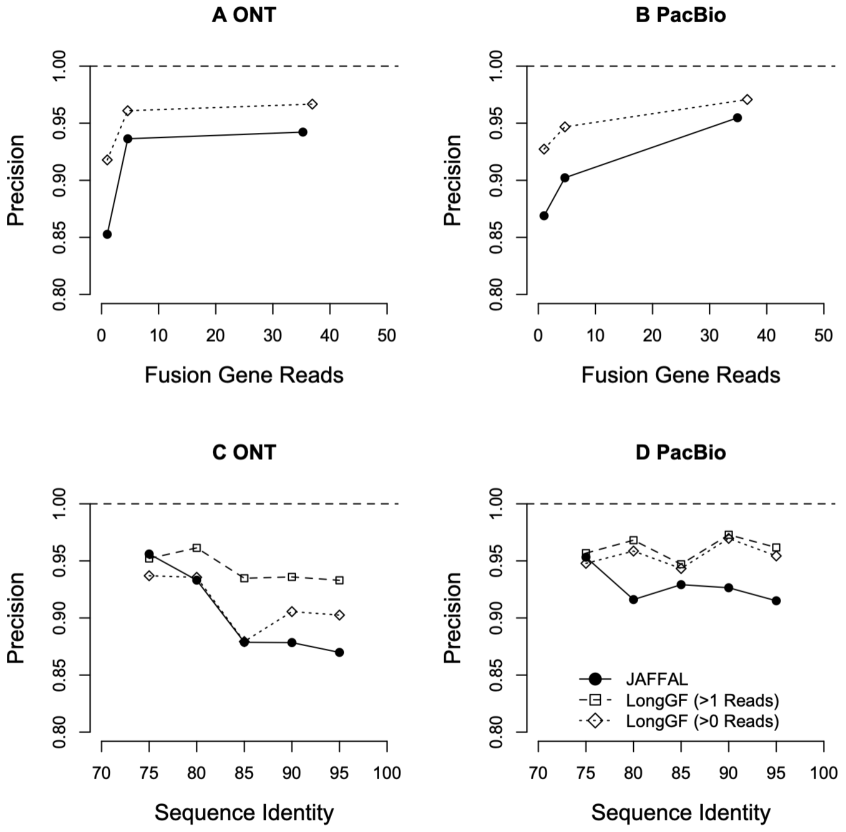

Fig. S6: Fusion finding precision on ONT and PacBio simulated fusion data without background. The fusion finding precision (true positives / positives) for JAFFAL and LongGF across a range of A) and B) fusion coverage levels and C) and D) read identity levels. The precision was within the range 0.85-0.95 across all coverage and read identity levels. Most false positives could be attributed to simulated fusions where one of the partner genes was misidentified (see manuscript for details).

Fusion	Tool	Predicted Breakpoint	Reads Support	Fusion Rank
BCR-ABL1	JAFFAL	chr22:23,182,239 - chr9:130,854,064	5	5th
	LongGF	chr22:23,182,237 - chr9:130,854,060	5	8th
RUNX1-RUNX1T1	JAFFAL	chr21:34,859,474 - chr8:92,017,363	8	1st
	LongGF	chr21:34,859,474 - chr8:92,017,365	11	2nd
IGH-CRLF2	JAFFAL	Not detected	-	-
	LongGF	Not detected	-	-

Table S7: Clinically relevant fusions detected in two patient samples by JAFFAL and LongGF. JAFFAL and LongGF show similar fusion ranking and read support. JAFFAL detects the exact breakpoints known from short-read sequencing

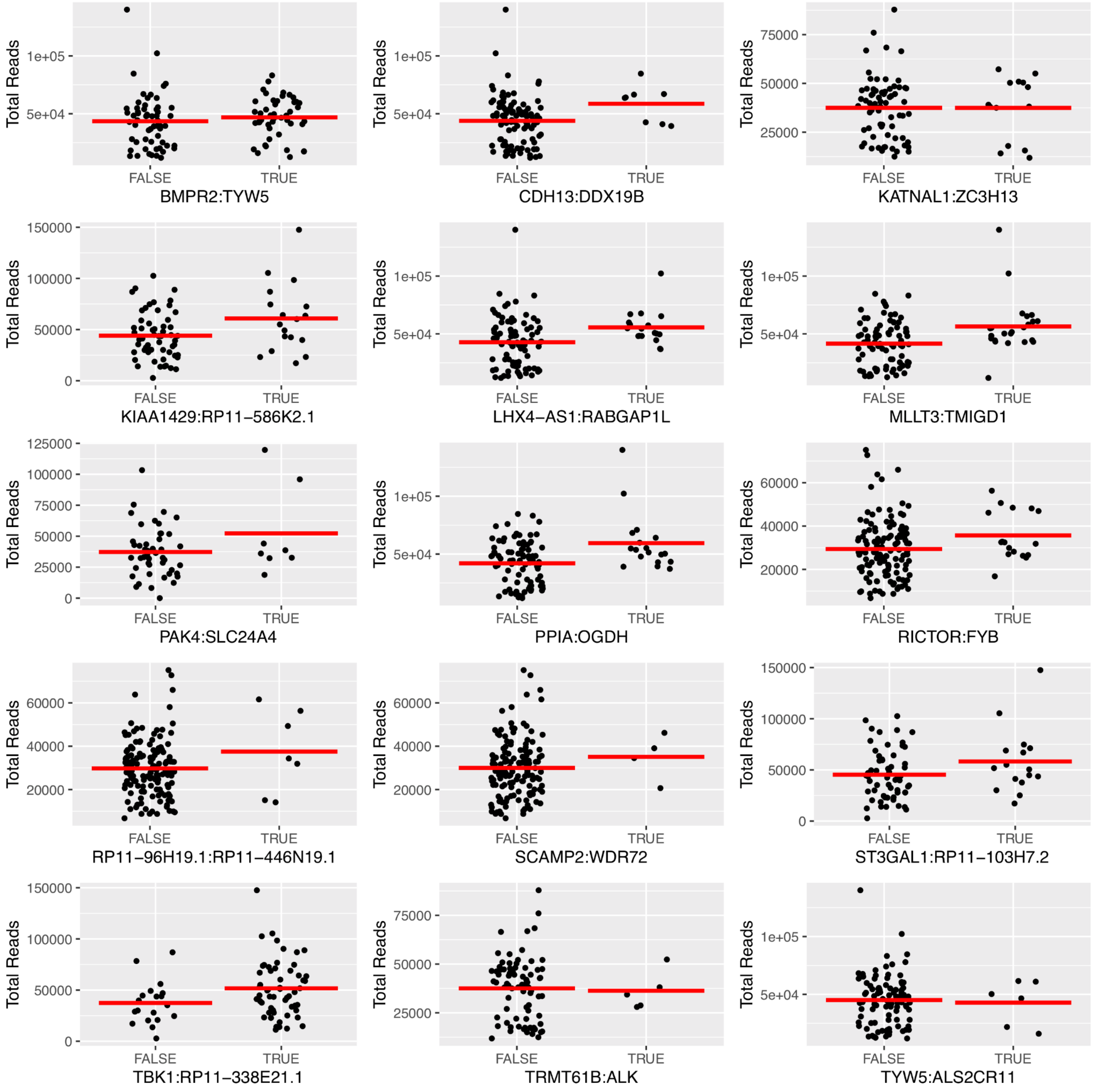

Fig. S7: Library size of single cells sequencing where fusions were or were not identified. For each fusion in the long read single cell sequencing data, we identified all cells in the corresponding gene expression cluster (see Manuscript). The total number of reads for each cell is shown (black) and mean (red bar) for cells where the given fusion was either identified (TRUE) or not (FALSE). Whether fusions could be identified in individual cells is likely to be a combination of total sequencing depth for the cell, heterogeneity in gene expression of the fusion and sampling. For most cells only a single fusion read was detected.

Cell line	Dataset	Fusion	Reads	Breakpoint Read	Breakpoint Classes	Orthogonal Evidence
H838	scRNA-Seq	BMPR2:TYW5:ALS2CR11	15	89:15	High:High	Both fusions seen in RNA and WGS from CCLE (Barretina et al, Nature, 2012)
H2228	scRNA-Seq	RP11-448A19.1:SND1:CFTR	4	18:4	Low:High	Both fusions seen in RNA from CCLE
MCF7	PacBio	TXLNG:SYAP1:RRM2	4	29:6	High:Low	-
H838	scRNA-Seq	XPR1:LHX4-AS1:RABGAP1L	2	2:64	High:High	-
MCF7	PacBio	BCAS4:BCAS3:REG4	2	1304:2	High:High	Both fusions seen in RNA from ENCODE (Davidson et al., Genome Med. 2014)
MCF7	SGNex	GBF1:MACROD2:C14orf132	1	13:2	High:High	Both fusions seen in matched Illumina data from SGNex
H838	scRNA-Seq	TRIP13:BMPR2:TYW5	1	1:89	TransSplicing:High	-
MCF7	SGNex	YY1:PPP1R12A:EVL	1	1:1	TransSplicing:TransSplicing	-
MCF7	SGNex	VMP1:BTBD1:YPEL5	1	1:1	TransSplicing:TransSplicing	-
MCF7	SGNex	RAD51B:CCDC170:EPB41L5	1	1:1	TransSplicing:TransSplicing	-
K562	SGNex	MPV17:TCERG1:CREBZF	1	1:1	TransSplicing:TransSplicing	-
MCF7	SGNex	IKZF2:NCOR1:SPATA33	1	1:1	TransSplicing:TransSplicing	-
MCF7	SGNex	CFL1:SLC4A7:URI1	1	1:1	TransSplicing:TransSplicing	-
MCF7	PacBio	COPS7B:AVL9:ZFYVE1	1	1:1	TransSplicing:TransSplicing	-

Table S9: Three-gene fusions identified by JAFFAL on cell line validation datasets. Reads indicated how many reads spanned the three genes. Breakpoint reads give the number of reads reported by JAFFAL for each individual fusion (separated by ":" in gene order). Breakpoint Classes indicated the classification of the individual fusions, as reported by JAFFAL.

(A)

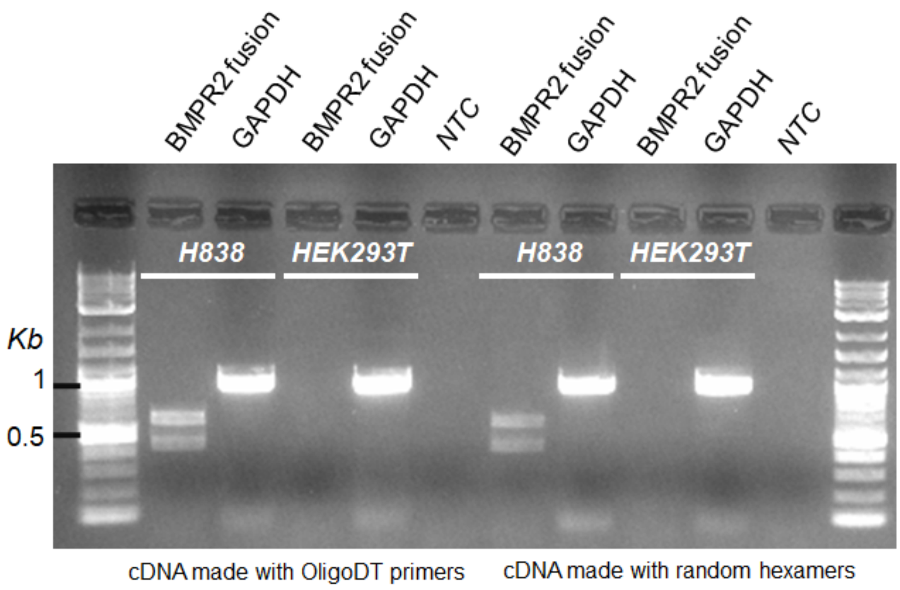

(B)

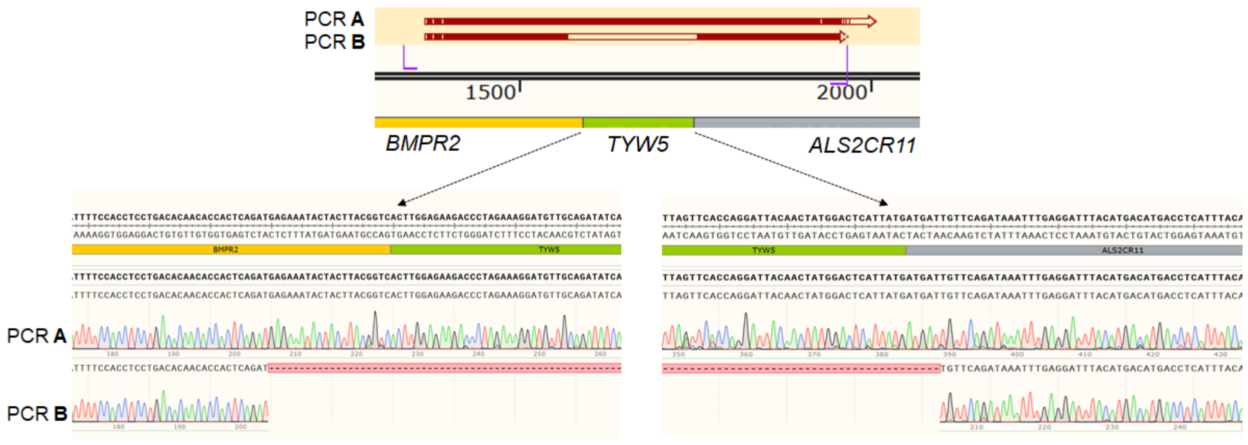

Fig. S8: Validation of BMPR2-TYW5-ALS2CR11 fusion. (A) PCR validation of BMPR2-TYW5-ALS2CR11 fusion in cDNA from H838 cells synthesized with OligoDT primers or random hexamers. cDNA from HEK293T cells was used as a negative control. (B) Sanger sequencing of the top band (PCR A) and lower band (PCR B) further confirmed these correspond to the three gene fusion BMPR2-TYW5-ALS2CR11 and it's two gene transcript, BMPR2-ALS2CR11, respectively.
