# Supplementary material for: JAFFAL: detecting fusion genes with long-read transcriptome sequencing
Source: Genome Biol. 2022 Jan 6;23:10. doi: 10.1186/s13059-021-02588-5 (PMC8739696; doi:10.1186/s13059-021-02588-5)
Supplement: Supplementary file 7 — Additional file 7:. Review history. [file 13059_2021_2588_MOESM7_ESM.docx]

Review History

**First round of review**

**Reviewer 1**

**Were you able to assess all statistics in the manuscript, including the appropriateness of statistical tests used?**

Yes, I was able to assess the statistics on the manuscript. Comments related to this are included in the review.

**Were you able to directly test the methods?**

No

**Comments to author:**

The authors describe JAFFAL, which is an extension of JAFFA to identify gene fusions from long-reads. The authors describe the analyses of simulated, bulk, and single cell datasets, present various benchmarking analyses, and a comparison with another method longGF. The method seems to work well and the results are promising. However, the analyses performed are not sufficient to show the advantages provided by JAFFAL and in general, to understand the benefits and limitations of using long-read sequencing to identify gene fusions.

I include below a list of questions and suggestions that may help improving the manuscript.

1 - In the abstract, the authors indicate that methods for fusion detection from long reads are lacking, however, there are three other methods available besides JAFFAL

AERON

https://www.biorxiv.org/content/10.1101/2020.01.27.921338v1

https://github.com/SchulzLab/Aeron

longGF

https://bmcgenomics.biomedcentral.com/articles/10.1186/s12864-020-07207-4

https://github.com/WGLab/LongGF

and

NanoFG

https://www.nature.com/articles/s41467-020-16641-7

https://github.com/SdeBlank/NanoFG

2 - the authors also say that JAFFAL "[provides] better accuracy than other long read fusion finders…" However, they only compare with longGF. They should include comparisons with the other two methods available.

3 - One of the limitations of JAFFAL, pointed out by authors, is that it relies on exon boundaries to assign the fusion break points. The method relies on this alignment to label fusions as high or low confidence. However, JAFFAL does not seem to provide a mean to control for true/false positives and to assess the reliability of potential new candidates. As a consequence, what is defined as high or low confidence may simply depend on the number of input reads, and there is no quantitative way to discriminate potential false positives.

4 - JAFFAL predictions are labelled as follows: "High Confidence" fusions are supported by two or more reads with breakpoints aligning to exon boundaries. "Low Confidence" fusions are also supported by two or more reads, but breakpoints do not align to exon boundaries. "Potential Trans-Splicing" events are supported by a single read, with breakpoints aligning to exon boundaries. This classification is not very informative to determine whether a novel fusion might be real or not:

4.1 - The read support will probably depend on the number of input reads. It could mean that a fusion label changes from trans-splicing to high-confidence simply by increasing the amount of sequencing. The authors should describe their metrics in terms of the read support found for the detected fusions and the number of input reads in each analyzed experiment. In many cases, the fusions are probably only found when multiple samples are pooled together.

4.2 - The classification is also artificial. Fusions can occur in the middle of an exon, or involve a new exon. Their definition is too dependent of the coordinates of the fusion, whereas there may be other more intrinsic metrics that could indicate how relevant / consistent the fusion is. One is the fusion allele frequency (see e.g. https://www.nature.com/articles/s41467-020-16434-y). Another one, more specific to this method, is the consistency of breakpoints across multiple reads.

5 - Some fusions occur in the middle of an exon. Are these also considered as potential high-confidence too?

6 - Chimeric reads in Nanopore cDNA sequencing may occur as a ligation artifact. These cases can be identified and fixed by locating internal adapters in the reads. The authors should pre-process the reads before analysing them for fusions, as these cases can be easily removed.

7- Are Trans-splicing fusions potentially a result of this experimental artifacts in cDNA libraries?

8- In the simulation analysis, the recall seems to increase with the % identity of the reads and with the number of supporting reads per fusion. The authors should also plot how the false positives depend on these two variables. This will help understand how reliable predictions in a new sample are, which is essential for fusion discovery.

9 - In the simulated datasets and in the other analyses, it is not made clear whether a fusion is considered found if the same gene-pair is found, or if both the gene-pair and the breakpoint is found. The authors should specify in each test what is being considered as a "found fusion". This will help understand the level of accuracy of the methods tested. For instance, are new breakpoints in known gene-pairs considered as novel? Or low confidence? What is the general accuracy in terms of breakpoint detection?

10 - The authors indicate that they were unsuccessful running AERON, one of the other three methods for fusion detection from long reads. However, the pre-prints for AERON and JAFFAL have one co-author in common. One would expect that this common co-author would facilitate that the comparisons of both methods is carried out. Furthermore, upon inspecting of AERON's github (https://github.com/SchulzLab/Aeron), there is an issue opened by the JAFFAL's first author related to running the snakemake pipeline for AERON with multiple Fastq files. The issue appears to have been resolved by the author in January 2021. It would be good if all authors from AERON and JAFFAL could make an effort to run both methods and compare them.

11 - Table 1 presents the fusions detected from dRNA and cDNA in NA12878 cells. They pooled a large number of replicates to obtain lots of reads. This raises the question of whether all those reads were necessary to detect the known fusion, and whether the potential artefacts or false positives could be filtered out in terms of the read support and/or other features. This large number of reads allows for a better analysis of how the fusion detection depends on the number of input reads and how the read support varies. The authors should also include the read support for the fusions detected and clarify whether these correspond to the same breakpoint in all cases, or variable breakpoints with same gene pairs.

12 - There is no discussion in the manuscript on how the detection and support of fusions (gene-pairs and breakpoints) depend on the number of input reads. This is a fundamental question in any experimental plan to identify fusions. It could be that the answer is that "one needs ">20M reads to detect a known fusion reliably". This would be useful to know, as it would mean that one MinION flowcell is not be sufficient to identify fusions. Without this information, it is not clear what the relevance of JAFFAL could be in a standard long-read sequencing experiment.

13 - Chimeric reads can be found by the identification of internal adapters. Have the authors tried to see whether the fusions reported with 1 read support are actually those with internal adapters? Also, how does this depend on the number of input reads? Do chimeras appear with higher read support in large input datasets, like the NA12878?

14 - In the analysis of multiple cancer cell lines (MCF7, HCT116,… ) the authors should provide the evaluation of TPs and FPs in terms of the read support and in terms of the number of input reads. Here, they are pooling again multiple samples and using a large number of reads as input. It could be that they detect fusions simply because there are lots of reads. It will be important to know whether there is a limitation in the detection of fusions if the number of input reads is below some threshold.

15 - The authors compare the fusions detected from long and short reads. Does the read support of these fusions correlate? This would be also essential to understand the depth of Nanopore sequencing required to detect fusions with a certain level of expression of allele frequency (see e.g. frequency (see e.g. https://www.nature.com/articles/s41467-020-16434-y). Does this comparison provide a clue of what thresholds / criteria to use to ensure a low false positive rate? How does this depend on the number of input reads?

16 - Overall the authors do not provide any means to control the false positives in the predictions in terms of e.g. read support, properties of the found breakpoints, etc. This would be desirable to make the discovery of fusions in new samples reliable.

17 - In the analysis of breakpoints in MCF7 cells, the authors seem to imply that there are multiple breakpoints found for the same gene pairs. Since all the data is from the same cells, one would expect that this variability is entirely due to errors of the data and/or processing. This could provide an opportunity to perform a more thorough assessment of the accuracy of breakpoint detection, and possibly on the reliability of the detection of novel fusions, especially if they have high read support. The authors suggest that this could be due to alternative splicing. However, they do not verify that, even though they have the appropriate data to do it. The authors should test whether the reads indicate alternative fusion transcripts, or whether the variation is due to the above-mentioned errors.

18 - The authors indicate that "Only 16% of fusions reported by JAFFAL were not seen by other

approaches, compared to 70% of LongGF's calls.". How does this depend on the actual read support? Also, is this just for gene-pairs? Or for breakpoints as well?

19 - In table 2 it seems that JAFFAL recovers many more known fusions than LongGF but at the cost of predicting many more potentially novel fusions. This could be a tradeoff between true positives and false positives. This analysis would be more useful if the authors could also provide the read support for these fusions and indicate whether there is a cutoff at which potential false positives can be kept to a minimum.

20 - In the analysis of the AML and B-ALL samples, JAFFAL detects the known fusions that involve existing exons. However, how many novel fusions were predicted? How many are potential false positives? What was the number of input reads in this case?

21 - identification of gene fusions at the single-cell level is not surprising, since the type of input data is exactly the same. The question that is not addressed by the authors in this case is how the potentially lower coverage in single-cells affect the identification. In this case, it would be interesting if the authors could provide information about how the number of reads per cell affect the identification of fusions, and whether that explains the detection differences. Also, is the same breakpoint generally found across single cells? At what coverage?

22 - The authors indicate that "For some cells, fusions were inconsistent with gene expression clustering". As gene expression patterns can separate cell types, the authors should be able to see whether these patterns are associated to the specific fusions detected. In this setting, one would expect that the same fusions would correlate with the same expression patterns, unless the number of reads is not sufficient for the detection of the fusion.

23 - In the assessment of computational resources, the authors indicate "All tools were given a maximum of four threads, apart from LongGF…". This is a strange statement, given that the authors only compare two tools.

23 - The comparison of computational resources between JAFFAL and LongGF should be done on the same number of threads. Otherwise, the comparison of run-time is not fair.

24 - In summary, although JAFFAL provides a framework to identify gene fusions from long reads, the authors did not provide information to assess whether it is feasible to identify gene fusions at breakpoint accuracy from Nanopore sequencing experiments, how many input reads are necessary to detect fusions, how this depends on the expression of the fusion, and how to discriminate candidate false positives from candidate true positives using read coverage or any other metric. The paper also provides limited comparison to other existing methods. In its current version, it is difficult to assess whether JAFFAL provides a useful tool to predict fusions from long read sequencing.

**Reviewer 2**

**Were you able to assess all statistics in the manuscript, including the appropriateness of statistical tests used?**

Yes: There do not appear have been any statistical tests used.

**Were you able to directly test the methods?**

No

**Comments to author:**

Davidson et al describe JAFFAL, an algorithm for detecting fusions in long reads. Long read fusion detection from long reads is currently an open problem with not many great tools. There is a broader and practical question of whether long reads are useful at all in fusion detection.

JAFFAL clusters alignments into breakpoint positions which are ranked on the basis of read support. In particular breakpoints that land in an exon or are only supported by a single read are discarded. The authors use simulations to to show incremental improvement against a previous algorithm (LongGF). Addition validation is done using "previously validated" fusions across several cell lines. The authors also apply their method to long read single cell RNA sequencing.

Overall, the results do not look impressive, either for this particular algorithm or more broadly long read RNA sequencing as a method to detect gene fusions. This is surprising a priori, but suggests that the data quality is poor and/or current methods are taking the wrong approach.

As with any SV paper, ground truth is a key challenge. Simulations usually do not represent the true data complexity. On the other hand, there is no great gold standard for real data.

Major comments

- The method does not seem innovative. A graph or assembly based approach would seem more likely to extract useful and biologically impactful fusion signal from the data.

- Validation tables (eg Table 2) don't make sense - for some samples the number of previously validated fusions detected by JAFFAL exceeds the total number of previously validated fusions.

- Table footnote "reciprocal translocations were counted as validated" is alarming, to say the least. Reciprocal translocations are not a validation. Order not only matters, the breakpoint location matters. This statement and the analytic choice made here calls into question the validity of the analysis. It also indicates that validation is not done at the breakpoint level, which again is incorrect.

- Improvement over LongGF seems incremental. Other long read methods including those that the authors discuss in the introduction are not benchmarked.

- What biological insight does this algorithm or long read RNA fusion detection provide over the state of the art? A long read fusion detection algorithm should provide some insight that short reads do not, such as full length fusion / splice isoform reconstruction

- Performance vs short reads seems surprisingly poor. Hard to see the utility of any method that does not exceed this very low bar.

- Validation should be done against an orthogonal dataset. For example, showing improvement of DNA-RNA correlation using whole genome sequencing, relative to competing methods and short read RNA-seq.

Minor comments:

- How were "previously validated" cell line fusions validated? How does this impact sensitivity and specificity estimates.

Precision and recall do not look impressive, and in particular not impressive relative to Illumina. Single cell ONT data applications

**Authors Response**

**Point-by-point responses to the reviewers’ comments:**

*Please find our revised manuscript for “JAFFAL: Detecting fusion genes with long read transcriptome sequencing”. A major update to this revision and our method is detection of complex multi-fusion transcripts which we feel further highlights the novelty of using long reads to detect fusion. We show that JAFFAL reports three-gene fusions and demonstrate that long reads reveal the complex splicing behaviour in such cases. We hope that this will address reviewer #2’s concerns about the significance and novelty of using long reads for fusion finding as this cannot generally be done with short read data. In the revised manuscript, we also give more information about support reads and clarify validation results as at the gene or breakpoint level.*

*Although we would love to benchmark against further methods, we were unable to run Aeron and we describe our efforts to achieve this in the comments to reviewer #1 below. NanoGF is a genome sequencing fusion finder and therefore not appropriate for RNA sequencing data. Indeed, the lack of alternative long read fusion finding methods demonstrates the important contribution of JAFFAL. We also argue that JAFFAL offers a significant advance over LongGF. We have modified our simulation of fusions to be more realistics by including background reads from the non-tumour reference cell line NA12878. With background reads, LongGF shows lower sensitivity than JAFFAL, consistent with our prior validation results using cancer cell lines, where LongGF missed 20% of the known fusions found by JAFFAL. As reviewer #2 indicates, reporting the correct fusion gene order and breakpoints is also important and LongGF does not do this reliably.*

Reviewer reports:

Reviewer #1: The authors describe JAFFAL, which is an extension of JAFFA to identify gene fusions from long-reads. The authors describe the analyses of simulated, bulk, and single cell datasets, present various benchmarking analyses, and a comparison with another method longGF. The method seems to work well and the results are promising. However, the analyses performed are not sufficient to show the advantages provided by JAFFAL and in general, to understand the benefits and limitations of using long-read sequencing to identify gene fusions.

I include below a list of questions and suggestions that may help improving the manuscript.

1 - In the abstract, the authors indicate that methods for fusion detection from long reads are lacking, however, there are three other methods available besides JAFFAL

AERON

https://www.biorxiv.org/content/10.1101/2020.01.27.921338v1

https://github.com/SchulzLab/Aeron

longGF

https://bmcgenomics.biomedcentral.com/articles/10.1186/s12864-020-07207-4

https://github.com/WGLab/LongGF

and

NanoFG

https://www.nature.com/articles/s41467-020-16641-7

https://github.com/SdeBlank/NanoFG

*We have replaced “lacking” with “few” in the abstract to clarify that other methods exist.*

2 - the authors also say that JAFFAL "[provides] better accuracy than other long read fusion finders..." However, they only compare with longGF. They should include comparisons with the other two methods available.

*We have tried extensively to benchmark against Aeron, on a number of different datasets and computing environments, but have been unsuccessful in running the program to completion. We have contacted the authors through their github repositories twice, most recently with no response. We note that the github repository has not been maintained for 9 months. We also requested their simulation data and results from Aeron’s preprint as a means to benchmark against JAFFAL, but this was not provided. Finally, we asked our common author to contact the Aeron authors and they confirmed that they would not be able to address the issues in time for this review.*

*NanoGF is a program designed to detect fusions in genome sequencing and not transcriptome sequencing data. Regardless, we tested whether it would work on transcriptome sequencing using our nanopore simulation of reads with 95% sequence identity and it was unable to detect any of the simulated fusions. As it was not designed for this type of data we do not believe it would be fair to NanoGF to include it in the comparative analysis. However, we have added a reference to NanoGF in the introduction of our manuscript to acknowledge its contribution to fusion detection.*

3 - One of the limitations of JAFFAL, pointed out by authors, is that it relies on exon boundaries to assign the fusion break points. The method relies on this alignment to label fusions as high or low confidence. However, JAFFAL does not seem to provide a mean to control for true/false positives and to assess the reliability of potential new candidates. As a consequence, what is defined as high or low confidence may simply depend on the number of input reads, and there is no quantitative way to discriminate potential false positives.

4 - JAFFAL predictions are labelled as follows: "High Confidence" fusions are supported by two or more reads with breakpoints aligning to exon boundaries. "Low Confidence" fusions are also supported by two or more reads, but breakpoints do not align to exon boundaries. "Potential Trans-Splicing" events are supported by a single read, with breakpoints aligning to exon boundaries. This classification is not very informative to determine whether a novel fusion might be real or not:

*Response to 3. and 4.: Fusion detection methods do not generally attempt to quantify false positive rates. False positives are generally real chimeric molecules that arise from true biology or library preparation, as we demonstrate in our manuscript. This means that false positive rates depend heavily on the type of sequencing, sample and library preparation as well as tissue type and are not easy to estimate. Mature fusion finding methods for short reads, e.g. Arriba, https://arriba.readthedocs.io/en/latest/interpretation-of-results/#confidence-scoring, use broad classes such as high, medium and low confidence. The reliability of our ranking, both between and within our discrete classes (high, low, potential trans-splicing) is demonstrated throughout the manuscript (ROC and tabulated classification results).*

4.1 - The read support will probably depend on the number of input reads. It could mean that a fusion label changes from trans-splicing to high-confidence simply by increasing the amount of sequencing. The authors should describe their metrics in terms of the read support found for the detected fusions and the number of input reads in each analyzed experiment. In many cases, the fusions are probably only found when multiple samples are pooled together.

*Indeed the number of supporting reads for a fusion will depend on the input reads, however it also depends on the expression level of the fusion gene. This is why we examined the fusion detection sensitivity in terms of the number of simulated fusion reads (Figure 2A). On real data, the true number of fusion reads is unknown. However, we show the total number of reads for all validation datasets in our manuscript as well as the impact of downsampling on fusion detection (Figure 3C).*

4.2 - The classification is also artificial. Fusions can occur in the middle of an exon, or involve a new exon. Their definition is too dependent of the coordinates of the fusion, whereas there may be other more intrinsic metrics that could indicate how relevant / consistent the fusion is. One is the fusion allele frequency (see e.g. https://www.nature.com/articles/s41467-020-16434-y). Another one, more specific to this method, is the consistency of breakpoints across multiple reads.

*Our results demonstrate that exon boundaries are an excellent metric for the prioritisation of fusion candidates and this is used extensively in many short read fusion callers. Fusion allele frequency can vary depending on the expression of the non-fused genes (if present) and tumour purity, therefore may not be as reliable for transcriptome data. We do use consistency of breakpoints across reads in our classification - “read support” is the number of reads with the same breakpoint (after adjusting for sequencing errors). We also note that multiple breakpoints can occur from a single genomic rearrangement due to alternative splicing.*

5 - Some fusions occur in the middle of an exon. Are these also considered as potential high-confidence too?

*Fusions with a breakpoint within an exon are reported as low confidence. The confidence levels are designed as a guide for the probability of a fusion being real. Fusions found with a break within an exon have a much higher probability of being a false positive (e.g. due to chimeras created during library preparation), but low confidence does not exclude a fusion as being real.*

6 - Chimeric reads in Nanopore cDNA sequencing may occur as a ligation artifact. These cases can be identified and fixed by locating internal adapters in the reads. The authors should pre-process the reads before analysing them for fusions, as these cases can be easily removed.

7-	Are Trans-splicing fusions potentially a result of this experimental artifacts in cDNA libraries?

*Response to 6. and 7.: The chimeras we discuss in the paper are different to those mentioned by the reviewer. There are no internal adapters in the chimeric reads we discuss and their hallmark is two partial transcripts which are joined, rather than two full transcripts. In the revised manuscript, we have applied PoreChop to the NA12878 (non-cancer) cDNA dataset to cut chimeras created from ligation and found little impact on the chimeras reported by JAFFAL (Supplementary Table 2). We also added clarification to the Section “JAFFAL’s fusion ranking is effective at separating false positives in non-tumor cell line data” of the manuscript, about these different types of chimeras.*

8-	In the simulation analysis, the recall seems to increase with the % identity of the reads and with the number of supporting reads per fusion. The authors should also plot how the false positives depend on these two variables. This will help understand how reliable predictions in a new sample are, which is essential for fusion discovery.

*False positive rates are difficult to assess from simulation as most false positives arise from background events where a true chimeric transcripts was sequenced, such as artifacts created during library preparation or trans-splicing. We examine the false positives in detail using a real non-cancer sample (NA12878). In the revised manuscript we show the number of supporting reads associated with these (Table 1 and Supplementary Table 1).*

9 - In the simulated datasets and in the other analyses, it is not made clear whether a fusion is considered found if the same gene-pair is found, or if both the gene-pair and the breakpoint is found. The authors should specify in each test what is being considered as a "found fusion". This will help understand the level of accuracy of the methods tested. For instance, are new breakpoints in known gene-pairs considered as novel? Or low confidence? What is the general accuracy in terms of breakpoint detection?

*We now provide our definition of found in the Results. This is also described in the methods section, “Comparison”. We discuss both the gene-level and breakpoint-level accuracy in Results. For example: “for 96% of fusions detected by JAFFAL, the exact breakpoint was reported, compared to just 2% from LongGF. However, almost all breakpoints were within 20bp of the simulated position for both tools.” in the simulation results. And the paragraph beginning “Breakpoint positions from JAFFAL were also consistent with short read data..” in the cell line results.*

10 - The authors indicate that they were unsuccessful running AERON, one of the other three methods for fusion detection from long reads. However, the pre-prints for AERON and JAFFAL have one co-author in common. One would expect that this common co-author would facilitate that the comparisons of both methods is carried out. Furthermore, upon inspecting of AERON's github (https://github.com/SchulzLab/Aeron), there is an issue opened by the JAFFAL's first author related to running the snakemake pipeline for AERON with multiple Fastq files. The issue appears to have been resolved by the author in January 2021. It would be good if all authors from AERON and JAFFAL could make an effort to run both methods and compare them.

*In addition to the issue we posted on AERON’s github page, we ran into other issues that were already reported by other users on github and had not been addressed. Also see our response to comments 1 and 2 above.*

11 - Table 1 presents the fusions detected from dRNA and cDNA in NA12878 cells. They pooled a large number of replicates to obtain lots of reads. This raises the question of whether all those reads were necessary to detect the known fusion, and whether the potential artefacts or false positives could be filtered out in terms of the read support and/or other features. This large number of reads allows for a better analysis of how the fusion detection depends on the number of input reads and how the read support varies. The authors should also include the read support for the fusions detected and clarify whether these correspond to the same breakpoint in all cases, or variable breakpoints with same gene pairs.

*We found an error in the number of reads we originally presented for the NA12878 dataset which has now been corrected to approx. 15 and 25 million reads. This depth demonstrates the robustness of the methods with respect to false discoveries. NA12878 is a negative control sample, in the sense that almost all fusions in this sample are false positives. It is therefore not possible to use this dataset to measure sensitivity. However, we now provide read support and breakpoint information in the Table as suggested.*

12 - There is no discussion in the manuscript on how the detection and support of fusions (gene-pairs and breakpoints) depend on the number of input reads. This is a fundamental question in any experimental plan to identify fusions. It could be that the answer is that "one needs ">20M reads to detect a known fusion reliably". This would be useful to know, as it would mean that one MinION flowcell is not be sufficient to identify fusions. Without this information, it is not clear what the relevance of JAFFAL could be in a standard long-read sequencing experiment.

*Also see our response to point 4.1. The sequencing depth required for fusion detection in RNA-Seq depends on the expression level of the fusion gene, which may vary even for the same fusion across individual patients. The number of input reads for each dataset we tested was already provided in the manuscript, but we now also show the range of supporting reads for relevant fusions and the full output from JAFFAL which includes supporting read information is provided in Supplementary Tables 1,5,6 and 8.*

13 - Chimeric reads can be found by the identification of internal adapters. Have the authors tried to see whether the fusions reported with 1 read support are actually those with internal adapters? Also, how does this depend on the number of input reads? Do chimeras appear with higher read support in large input datasets, like the NA12878?

*We now address this in the revised manuscript and Supplementary Tables 2 and 3. See also response to comments 6 and 7.*

14 - In the analysis of multiple cancer cell lines (MCF7, HCT116,... ) the authors should provide the evaluation of TPs and FPs in terms of the read support and in terms of the number of input reads. Here, they are pooling again multiple samples and using a large number of reads as input. It could be that they detect fusions simply because there are lots of reads. It will be important to know whether there is a limitation in the detection of fusions if the number of input reads is below some threshold.

*Also see our response to similar comments above. Figure 3C demonstrates the impact of input read depth on fusion detection. Many fusions are still detected with only 1 GB of sequencing data. The concerns raised in this and similar comments related to the utility of RNA sequencing for fusion detection more generally. This has been addressed through a substantial body of research over the last decade in the context of short read data (e.g. Edgren et al. Genome Biology 2011, Kumar et al., Gao et al. Wiley Interdiscip Rev RNA 2016, .Cell Rep., 2018).*

15 - The authors compare the fusions detected from long and short reads. Does the read support of these fusions correlate? This would be also essential to understand the depth of Nanopore sequencing required to detect fusions with a certain level of expression of allele frequency (see e.g. frequency (see e.g. https://www.nature.com/articles/s41467-020-16434-y). Does this comparison provide a clue of what thresholds / criteria to use to ensure a low false positive rate? How does this depend on the number of input reads?

*Please see our response to prior comments.*

16 - Overall the authors do not provide any means to control the false positives in the predictions in terms of e.g. read support, properties of the found breakpoints, etc. This would be desirable to make the discovery of fusions in new samples reliable.

*See also our response to comment 3 and 4. We provide a ranking for gene prioritisation and out classification is a way of controlling for the false positives.*

17 - In the analysis of breakpoints in MCF7 cells, the authors seem to imply that there are multiple breakpoints found for the same gene pairs. Since all the data is from the same cells, one would expect that this variability is entirely due to errors of the data and/or processing. This could provide an opportunity to perform a more thorough assessment of the accuracy of breakpoint detection, and possibly on the reliability of the detection of novel fusions, especially if they have high read support. The authors suggest that this could be due to alternative splicing. However, they do not verify that, even though they have the appropriate data to do it. The authors should test whether the reads indicate alternative fusion transcripts, or whether the variation is due to the above-mentioned errors.

*Alternative splicing of fusons is well documented (e.g. Kangaspeska, PLoS One, 2012) and breakpoint detection accuracy is assessed to be near perfect in our simulation. In our revised manuscript we include data which validates alternative splicing of a three-gene fusion we find in the H838 cell line.*

18 - The authors indicate that "Only 16% of fusions reported by JAFFAL were not seen by other approaches, compared to 70% of LongGF's calls.". How does this depend on the actual read support? Also, is this just for gene-pairs? Or for breakpoints as well?

*We have reworded to clarify: ”Only 16% of fusion genes reported by JAFFAL as high or low confidence were not seen by other approaches, compared to 70% of LongGF’s calls (>1 read support)”.*

19 - In table 2 it seems that JAFFAL recovers many more known fusions than LongGF but at the cost of predicting many more potentially novel fusions. This could be a tradeoff between true positives and false positives. This analysis would be more useful if the authors could also provide the read support for these fusions and indicate whether there is a cutoff at which potential false positives can be kept to a minimum.

*The trade of between true positives and potential false positives is shown in the ROC curve in Figure 3A and 3B. Here the different cut-off levels we suggest are defined by the high confidence, low confidence and potential trans-splicing levels which are a combination of read support and breakpoint position relative to exon boundaries. The ROC curve demonstrates that high and low confidence fusions have a high rate of true positives.*

20 - In the analysis of the AML and B-ALL samples, JAFFAL detects the known fusions that involve existing exons. However, how many novel fusions were predicted? How many are potential false positives? What was the number of input reads in this case?

*We have now added the number of high confidence calls in the section on patient samples. Our prior validation results suggest that the majority of these are likely to be true fusions. The number of input reads (8 and 13 million) is provided in the introductory paragraph to this section.*

21 - identification of gene fusions at the single-cell level is not surprising, since the type of input data is exactly the same. The question that is not addressed by the authors in this case is how the potentially lower coverage in single-cells affect the identification. In this case, it would be interesting if the authors could provide information about how the number of reads per cell affect the identification of fusions, and whether that explains the detection differences. Also, is the same breakpoint generally found across single cells? At what coverage?

*We now provide information about the number of reads for cells where fusions are or are not identified (Supplementary Figure 5) and the range of reads supported per cell when they are found, which is generally low (median=1 read). It is difficult to separate how low coverage impacts identification from heterogeneity in the expression of fusion genes between cells. We also note that fusions do not need to be identified in all cells within a gene expression cluster, only a subset, to associate fusions with expression profiles.*

22 - The authors indicate that "For some cells, fusions were inconsistent with gene expression clustering". As gene expression patterns can separate cell types, the authors should be able to see whether these patterns are associated to the specific fusions detected. In this setting, one would expect that the same fusions would correlate with the same expression patterns, unless the number of reads is not sufficient for the detection of the fusion.

*We show that fusions do correspond to gene expression patterns in Figure 4A and B. However, there are some fusion reads that appear to be misassigned to cells likely due to errors in barcoding of the reads. We have reworded the sentence to clarify “Some fusions were detected in the wrong cell line cluster.”.*

23 - In the assessment of computational resources, the authors indicate "All tools were given a maximum of four threads, apart from LongGF...". This is a strange statement, given that the authors only compare two tools.

*We have reworded this sentence.*

23 - The comparison of computational resources between JAFFAL and LongGF should be done on the same number of threads. Otherwise, the comparison of run-time is not fair.

*LongGF being single threaded is a limitation of the method and we aimed to run both programs in a way that was similar to common usage, hence multiple threads. Despite the number of threads, we show that LongGF is substantially faster and uses less RAM than JAFFAL. We have reworded the text to highlight this better.*

24 - In summary, although JAFFAL provides a framework to identify gene fusions from long reads, the authors did not provide information to assess whether it is feasible to identify gene fusions at breakpoint accuracy from Nanopore sequencing experiments, how many input reads are necessary to detect fusions, how this depends on the expression of the fusion, and how to discriminate candidate false positives from candidate true positives using read coverage or any other metric. The paper also provides limited comparison to other existing methods. In its current version, it is difficult to assess whether JAFFAL provides a useful tool to predict fusions from long read sequencing.

*We hope we have addressed these points in our response to prior comments.*

Reviewer #2: Davidson et al describe JAFFAL, an algorithm for detecting fusions in long reads. Long read fusion detection from long reads is currently an open problem with not many great tools. There is a broader and practical question of whether long reads are useful at all in fusion detection.

JAFFAL clusters alignments into breakpoint positions which are ranked on the basis of read support. In particular breakpoints that land in an exon or are only supported by a single read are discarded. The authors use simulations to to show incremental improvement against a previous algorithm (LongGF). Addition validation is done using "previously validated" fusions across several cell lines. The authors also apply their method to long read single cell RNA sequencing.

Overall, the results do not look impressive, either for this particular algorithm or more broadly long read RNA sequencing as a method to detect gene fusions. This is surprising a priori, but suggests that the data quality is poor and/or current methods are taking the wrong approach.

As with any SV paper, ground truth is a key challenge. Simulations usually do not represent the true data complexity. On the other hand, there is no great gold standard for real data.

Major comments

- The method does not seem innovative. A graph or assembly based approach would seem more likely to extract useful and biologically impactful fusion signal from the data.

*Although simulation can not represent the true complexity of data, our simulation results do indicate that JAFFAL identified fusions with excellent recall (Figure 2A and Supplementary Figures 2 and 3) with the major limitation being sequencing noise rather than the algorithm. Short read fusion finding suggests that assembly based approaches are less sensitive due to the need for higher coverage (Haas et. al, Genome Biology, 2019) and this is also likely to be the case for long read sequencing.*

- Validation tables (eg Table 2) don't make sense - for some samples the number of previously validated fusions detected by JAFFAL exceeds the total number of previously validated fusions.

*This table has now been updated.*

- Table footnote "reciprocal translocations were counted as validated" is alarming, to say the least. Reciprocal translocations are not a validation. Order not only matters, the breakpoint location matters. This statement and the analytic choice made here calls into question the validity of the analysis. It also indicates that validation is not done at the breakpoint level, which again is incorrect.

*The reciprocal fusion was included in the table for two reasons:*

*1.	LongGF’s sensitivity would be significantly reduced if we only considered correct fusion gene order and this did not seem fair when comparing it with JAFFAL (which reports the correct order)*

*2.	Translocations are often balanced. If a reciprocal fusion is identified, then this is highly likely to be a true positive (despite being unvalidated).*

*However, to avoid confusion we no longer consider reciprocal fusions as true positives if the non-reciprocal (correct gene order) is also identified. We have also added the sentence “All fusions were reported with genes in the correct 5’ and 3’ order for JAFFAL compared to 68% for LongGF” to emphasize this limitation of LongGF.*

*Although validation of breakpoint positions is important, and we do present results on breakpoint accuracy in the manuscripts, we disagree that validation must be performed at breakpoint level exclusively. LongGF for example, reports the incorrect breakpoint for many fusions (see simulation results). This does not invalidate its gene-level detection which arguably is more important for clinical screening. We also note that fusion genes can be alternatively spliced and only some splice variants have been previously validated.*

- Improvement over LongGF seems incremental. Other long read methods including those that the authors discuss in the introduction are not benchmarked.

*We show that JAFFAL offers better accuracy than LongGF for both detection of fusion gene pairs as well as predicting breakpoints if found. For example, we show that around 20% of the known fusions detected by JAFFAL in cell line data are missed by LongGF, even after allowing LongGF to report fusions with 1 read support and with at least twice the rate of false positives compared to JAFFAL (Figure 3A and 3B). We also note that JAFFAL reports fusion genes in order, unlike LongGF and we now mention in the text.*

*In the new manuscript we have updated our simulation to include real background reads from non-fused genes which provides a more realistic dataset for benchmarking. We find that LongGF’s sensitivity is reduced in the presence of background compared to our prior simulation. These new simulation results are consistent with our cancer cell line benchmarking and support JAFFAL having superior sensitivity.*

*We address benchmarking against other tools in our response to reviewer 1’s comment 2.*

- What biological insight does this algorithm or long read RNA fusion detection provide over the state of the art? A long read fusion detection algorithm should provide some insight that short reads do not, such as full length fusion / splice isoform reconstruction

*To address this we have added a new section “JAFFAL detects three-gene fusions”, which looks at reads that include multiple breakpoint events. We added functionality to detect these complex fusions and now show that complex fusion isoforms can be recovered using long read sequencing (Figure 4C).*

- Performance vs short reads seems surprisingly poor. Hard to see the utility of any method that does not exceed this very low bar.

*We would argue that short read fusion finding methods are exceptionally good at detecting fusions which are expressed and not all previously validated fusions are detected even by short reads. Many challenges to fusion finding are independent of sequencing type such as low expression of the fusion gene, fusions to promoters with no transcription of the breakpoint and backgrounds from true chimeric molecules.*

*The utility of long read fusion finding over short reads is in combination with other advances offered by long reads. For example, faster diagnostics, obtaining full transcript sequences, use with high throughput single cell platforms etc. In addition, the accuracy of long read sequencing is likely to increase rapidly making it more widely utilised and therefore having the correct analysis tools will be important.*

- Validation should be done against an orthogonal dataset. For example, showing improvement of DNA-RNA correlation using whole genome sequencing, relative to competing methods and short read RNA-seq.

*We used orthogonal datasets, such as fusions from whole genome sequencing, Sanger sequencing and fusions detected in short-read sequencing from CCLE to define true positives in our benchmarking. Higher sensitivity therefore implies higher correlation with the results from these other sources.*

Minor comments:

- How were "previously validated" cell line fusions validated? How does this impact sensitivity and specificity estimates.

*We now clarify this in the text and provide a list of fusions with literature sources in Supplementary Table 4.*

Precision and recall do not look impressive, and in particular not impressive relative to Illumina. Single cell ONT data applications

*Short read fusion finding methodology has had a decade of development (e.g. Creason et al. Cell Systems, 2021), we would not expect long read fusion detection to exceed its performance in particular in light of the high error rate which impacts sensitivity (Figure 2).*

**Second round of review**

**Reviewer 1**

The authors have made multiple changes in the manuscript. Unfortunately, those edits are not highlighted in a different color, which makes it very hard to identify where these edits were. The authors have also failed to address some of the question initially posed.

The authors have now clarified how the breakpoints are considered as "found" in the simulated data: "Fusions were considered detected if a breakpoint was reported within 1kbp euclidean distance of the simulated breakpoint." This is a very big distance and too lenient. If this is the criterion used throughout the manuscript, the authors should also indicate what is the average distance found in the fusions reported to be detected in other samples.

In the original submission, the authors were asked to study the potential false positives from the simulated data before the introduced the background. The simulated data is the perfect setting to estimate the rate of false positives, since only the introduced fusions should be found. The authors did not respond to this query. Instead, they argued that false positives may be due to chimeric transcripts from the background reads introduced. However, these backgrounds were introduced in the revision and were not present in the original version. The authors have not addressed this issue and it remains unclear what the expected false positive rate of their method is.

The authors claim that "Fusion detection methods do not generally attempt to quantify false

positive rates.". This statement is problematic, as every computational detection method should report false positives. Without such estimates, there is no clarity of the expected behaviour in genome-wide or transcriptome-wide predictions in new samples, especially with a large number of input reads.

The authors also claim "False positives are generally real chimeric molecules that arise from true biology

or library preparation, as we demonstrate in our manuscript". This is again a problematic statement. They only show that there are fusions in ONT cDNA reads that do not appear in ONT RNA reads, which can be due to various possibilities, including the fact that internal priming of the cDNA library preparation could lead to chimeric artifacts. However, these additional fusions could also appear since ONT cDNA sequencing generally produce more reads and could present PCR amplification biases, and the analysis presented do not distinguish the detection power / accuracy from the number of input reads. Moreover, the authors do not present evidence that would support that new fusions detected from direct RNA sequencing are real chimeric molecules. This case is especially relevant, as with direct RNA sequencing library preparation is unlikely to produce chimeric reads.

JAFFAL defines "Potential Trans-Splicing" events as those "supported by a single read,

with breakpoints aligning to exon boundaries". This is ambiguous, as according to this definition, for any real fusion there will be some sequence depth at which it will be classified as "Potential Trans-Splicing" event, and some other (most likely higher) sequencing depth at which it could be classified as "high confidence". This makes this distinction very ambiguous, especially when discussing the accuracy of fusion detection.

The authors detect >300 such fusions in ONT direct RNA sequencing reads (Table 1). This analysis suggests that using 14M ONT RNA input reads, they get >300 false positives. However, they dismiss those cases by considering them compatible with what they observed in short reads. Here they cite the JAFFA paper, but this is not clearly stated in that paper. The authors do not address the issue of the potential false positives in a rigorous way. The cell line dataset is also clearly not appropriate to assess false positives, as they do find fusions that they cannot determine whether they are real or not. This goes back to my original question of how to separate potential false positives from the likely candidates and whether they could assess that in the simulated data or any controlled dataset.

Application of JAFFAL to ONT data from various cell lines does not clarify this aspect any further.

Table 2 reports that one needs between 31M and 44M input ONT reads to identify half the known fusions, while reporting between 600-2400 other novel fusions, some of those at high confidence. On the other hand, the authors claim in the abstract that "fusions can be accurately detected in long-read data with JAFFAL" but the data is not clear and the conditions are highly unrealistic. According to their estimates, one would need at least 30M reads for an estimated 50% sensitivity and low precision. This number of reads is also probably highly unrealistic. How many sequencing runs did they have to merge to achieve this detection? This comes back to the question of whether the detection power simply depends on the number of input reads. Is it then realistic to expect fusion detections from standard nanopore / Pacbio sequencing runs? JAFFAL does not appear to have been tested in standard experimental conditions.

JAFFAL shows low precision in the MCF7 data (Figure 3C) and it has to go down below 0.1 precision (i.e. >90% of the fusion calls are not correct) to achieve ~60% sensitivity. Although this dataset is not the best one to assess precision, the results do not support the authors' claim: "Taken together, these results suggest JAFFAL is highly accurate".

The authors also claim "We show that fusions can be accurately detected in long read data with JAFFAL,

providing better accuracy than other long read fusion finders". This sentence should say "than another long read fusion finder", as the comparison is only made with LongGF. Additionally, given the high rate of potential false positives or the lack of a strategy to confidently separate true and separate positives, it is not clear that fusion detection with long-reads is "accurate".

The authors claim "We show JAFFAL is the most accurate fusion finder available for noisy long read data, allowing fusions to be detected in long read data with similar accuracy as short reads." This should be toned down, as it is the best of two, and in the comparisons shown in the current version of the manuscript. In the previous version, in the simulated ONT data without background, LongGF faired quite well.

Perhaps the manuscript is too focused on presenting a novel and accurate method. However, the results do not fully support the authors' claims. On the other hand, the manuscript is possibly presenting the most comprehensive study to date on gene-fusions from long read data. This could provide an opportunity to rewrite the article as a more realistic perspective of the limitations and opportunities of long read sequencing to study gene fusions.

**Reviewer 2**

Davidson et al present a revised version of their manuscript "JAFFAL ... ". The revision and rebuttal does not address the key concerns raised during initial review, including algorithmic innovation and qualitative improvement over the state of the art. As mentioned before, JAFFAL's improvement over existing methods (LongGF) seems incremental and overall performance is poor with no clear value add over short reads. Provided examples (multi-gene fusion) seem anecdotal, of uncertain significance, and also detectable in short read paired end RNA-seq. There are still no fundamental insights given by JAFFAL that previous methods (including RNA-seq) miss. The suggested analysis of splice isoforms and fusions was not attempted. The analysis of RNA fusions with ONT data is indeed challenging due to sequencing noise. However, the read lengths are also 1-2 orders of magnitude longer than Illumina. So there should be novel insights. It's not clear whether JAFFAL (or LongGF for that matter) have solved the problem of analyzing these data. Perhaps simple pipelines like JAFFAL and LongGF that take alignments as-is and apply filters can only get so far. More innovative approaches to nanopore alignment, assembly, or raw signal processing may thus be needed to achieve an advancement the state of the art in transcriptomics, i.e. short read RNA-seq. Otherwise, what is the incentive to use ONT to study RNA fusions? If the results in this paper represent the analytic ceiling, they actually indicate no benefit to using ONT for RNA fusion detection. Major improvements in the fidelity of nanopore may be required to make ONT practically useful for fusion detection. However, if / when that happens - new algorithms will be likely needed to process that data. Indeed the analytic state of the art took years to mature for RNA-seq, However, prior to Illumina, it was impossible to detect fusions or splice isoforms de novo genome-wide. Each step in method development raised the bar in whole transcriptome analysis, which is now indeed higher than 10-15 years ago. Though JAFFAL may be an adequate pipeline to analyze ONT RNA-seq, it does not seem to significantly advance the state of the art in transcriptome analysis.

**Authors Response**

**Point-by-point responses to the reviewers’ comments:**

*Please find our revised manuscript with changes highlighted in red and our response to review*

*1’s remaining concerns, which focus on assessment of false positives.*

*In the revised manuscript we:*

*-	performed a new simulation of background events where no fusions are present (negative simulation) to assess the false positive rate due to non-biological artifacts. JAFFAL reports very few false positives on this dataset, demonstrating that the observed events found in the non-tumour cell line data are unlikely to be artifacts of the data processing.*

*-	address the question of how the number of false positives change with sequencing depth, by including a new Supplementary Figure (Supplementary Figure 2). Here, we downsampled the non-tumour datasets (simulation and cell line) and found that background such as chimeras created from library preparation and trans-splicing scale with the number of reads, however the number of high confidence calls from JAFFAL remains low.*

*-	We have added back in a short discussion on the false positives found in the fusion simulation which was present in our original submission, but removed in the last revision. We have also included a new Supplementary Figure (Supplementary Figure 6) which looks at fusion detection precision in the fusion simulation by coverage and read identify levels.*

*-	Finally we have reworked Figure 3C which previously showed the “Precision” and “Recall” of fusing finding for ONT data compared to short reads for the MCF-7 cell line. As precision and recall can not be accuracy known on this data we now plot the number of fusions which have or have not been previously validated, consistent with the other subfigures in Figure 3.*

*-	Rearranged the sections to now present our assessment of false positive rate prior to sensitivity, for flow of reading.*

*Response to reviewer report:*

*Reviewer #1: The authors have made multiple changes in the manuscript. Unfortunately, those edits are not highlighted in a different color, which makes it very hard to identify where these edits were. The authors have also failed to address some of the question initially posed.*

*In the revised manuscript we now highlight the new changes in red. This version is included as an additional data file as requested by the editor. We hope we have addressed remaining concerns in the new manuscript and with our responses below.*

*The authors have now clarified how the breakpoints are considered as "found" in the simulated data: "Fusions were considered detected if a breakpoint was reported within 1kbp euclidean distance of the simulated breakpoint." This is a very big distance and too lenient. If this is the criterion used throughout the manuscript, the authors should also indicate what is the average distance found in the fusions reported to be detected in other samples.*

*This is similar to a comment from review 2 in the previous reviews. It was addressed in our prior revision by stating in Results: “Fusion genes reported by JAFFAL and LongGF were compared to those previously validated using gene identifiers. When a fusion had multiple breakpoints, we assigned the fusion gene the classification of its highest rank breakpoint.” and in Methods: “For cell lines data we matched fusions to those previously validated (Supplementary Table 2) and between fusion finders using gene names. Novel breakpoints within known fusion gene pairs were considered true positives. Unless stated otherwise, comparisons were performed at the fusion gene-level, meaning fusions with multiple breakpoints were counted as a single true or false positive, and given the ranking of their highest ranked breakpoint.”*

*We do not use exact breakpoint to assess true positives on cell line data because:*

*-	Exact breakpoint positions are not reported for all previously validated fusions in the literature*

*-	Fusion genes can have multiple breakpoint due to splicing*

*-	Imprecise breakpoint determination, does not invalidate gene-level detection which arguably is more important for clinical screening.*

*Instead we assess the breakpoint position accuracy separately using 1) our fusion simulation, 2) consistency between breakpoints reported for MCF-7 in long and short read data and 3) on the patient samples.*

In the original submission, the authors were asked to study the potential false positives from the simulated data before the introduced the background. The simulated data is the perfect setting to estimate the rate of false positives, since only the introduced fusions should be found. The authors did not respond to this query. Instead, they argued that false positives may be due to chimeric transcripts from the background reads introduced. However, these backgrounds were introduced in the revision and were not present in the original version. The authors have not addressed this issue and it remains unclear what the expected false positive rate of their method is.

*We have now performed a negative simulation of unfused transcripts and provide details of this in the manuscript. JAFFAL identified just 9 fusions (4 high confidence) from 9 million simulated reads (~250 thousand transcripts). We feel that this new negative simulation gives a better indication of the false positive rate than the fusion simulation. The fusion simulation does not include unfused transcripts and is a small number of reads (88 thousand). However, we have also expanded our discussion of the false positives in the fusion simulation, which are primarily due to the misidentification of one fusion partner. We hope these new results alleviate concerns about false positives and help to justify the confidence system we use to separate chimeric and trans-splicing events from fusion genes.*

The authors claim that "Fusion detection methods do not generally attempt to quantify false positive rates.". This statement is problematic, as every computational detection method should report false positives. Without such estimates, there is no clarity of the expected behaviour in genome-wide or transcriptome-wide predictions in new samples, especially with a large number of input reads.

*Our observed false positives on the negative simulation are now shown in Supplementary Figure 2. The false positive rate is extremely low (<0.001) given the number of genes we are testing. The precision for the fusion simulation is also now shown in Supplementary Figure 6.*

The authors also claim "False positives are generally real chimeric molecules that arise from true biology or library preparation, as we demonstrate in our manuscript". This is again a problematic statement. They only show that there are fusions in ONT cDNA reads that do not appear in ONT RNA reads, which can be due to various possibilities, including the fact that internal priming of the cDNA library preparation could lead to chimeric artifacts. However, these additional fusions could also appear since ONT cDNA sequencing generally produce more reads and could present PCR amplification biases, and the analysis presented do not distinguish the detection power / accuracy from the number of input reads. Moreover, the authors do not present evidence that would support that new fusions detected from direct RNA sequencing are real chimeric molecules. This case is especially relevant, as with direct RNA sequencing library preparation is unlikely to produce chimeric reads.

*We hope we have addressed these concerns with our new simulation and Supplementary Fig 2 which show the rate of these false positives as a function of library size. We show that almost no fusions are reported from a negative simulation, suggesting that those seen in real data are likely to be created from true chimeric molecules and these have been documented previously e.g. Houseley and Tollervey, PLoS One. 2010 Aug 18;5(8):e12271 and Jia et al., Trends Cancer. 2016 Sep; 2(9): 475–484 and are well known sources of background in fusion finding that are filtered out, e.g. Creason et al., Cell Syst. 2021 Aug 18;12(8):827-838.e5. We also note that the chimeric artifacts we observe in cDNA libraries are also seen for direct cDNA sequencing (Supplementary Figure 3).*

*The detection power for fusion finding depends on fusion gene expression levels as well as the number of input reads - this is why we assess fusion sensitivity as a function of read coverage (Figure 2), which is a metric incorporating both gene expression and library size.*

JAFFAL defines "Potential Trans-Splicing" events as those "supported by a single read, with breakpoints aligning to exon boundaries". This is ambiguous, as according to this definition, for any real fusion there will be some sequence depth at which it will be classified as "Potential Trans-Splicing" event, and some other (most likely higher) sequencing depth at which it could be classified as "high confidence". This makes this distinction very ambiguous, especially when discussing the accuracy of fusion detection.

*Unlike fusions, trans-splicing events appear to be stochastic. With more reads, we detect more distinct trans-splicing events, rather than more reads for the same events. Hence, we do not expect trans-splicing events to shift classification with deeper sequencing. The low number of high confidence fusions seen in NA12878 supports this (Table 1., Supplementary Figure 2).*

*We agree that true fusions can be reported as potential trans-splicing due low library size or tumour purity, and this is why JAFFAL reports these events despite the extreme majority being background events.*

The authors detect >300 such fusions in ONT direct RNA sequencing reads (Table 1). This analysis suggests that using 14M ONT RNA input reads, they get >300 false positives. However, they dismiss those cases by considering them compatible with what they observed in short reads. Here they cite the JAFFA paper, but this is not clearly stated in that paper. The authors do not address the issue of the potential false positives in a rigorous way. The cell line dataset is also clearly not appropriate to assess false positives, as they do find fusions that they cannot determine whether they are real or not. This goes back to my original question of how to separate potential false positives from the likely candidates and whether they could assess that in the simulated data or any controlled dataset.

*We hope we have now addressed this using the new negative simulation and downsampling analysis (Supplementary Figure 2). The cell line data we used to assess false positives was NA12878, which is absent from fusions apart from two found in the healthy population which we describe. NA12878 has been extensively profiled with genome sequencing and known to have a regular diploid genome without extensive structural rearrangements.*

Application of JAFFAL to ONT data from various cell lines does not clarify this aspect any further. Table 2 reports that one needs between 31M and 44M input ONT reads to identify half the known fusions, while reporting between 600-2400 other novel fusions, some of those at high confidence. On the other hand, the authors claim in the abstract that "fusions can be accurately detected in long-read data with JAFFAL" but the data is not clear and the conditions are highly unrealistic. According to their estimates, one would need at least 30M reads for an estimated 50% sensitivity and low precision. This number of reads is also probably highly unrealistic. How many sequencing runs did they have to merge to achieve this detection? This comes back to the question of whether the detection power simply depends on the number of input reads. Is it then realistic to expect fusion detections from standard nanopore / Pacbio sequencing runs? JAFFAL does not appear to have been tested in standard experimental conditions.

*We demonstrate that JAFFAL identified fusions over a range of library sizes:*

*-	150 thousand - 3 million PacBio reads (cell lines)*

*-	31-44 million ONT reads (cell lines)*

*-	9 and 13 million ONT reads (patient)*

*-	800-148 thousand ONT reads (single cells)*

*Although 30 million reads is deeply sequenced, this is well within the depth generated by one flow cell of a PromethION. Our patient ALL sample (13 million reads) was generated from a single MinION flow cell. We also note that long read RNA sequencing generally produces reads shorter than two thousand bases on average, therefore a much higher number of reads is expected than for genomic sequencing.*

JAFFAL shows low precision in the MCF7 data (Figure 3C) and it has to go down below 0.1 precision (i.e. >90% of the fusion calls are not correct) to achieve ~60% sensitivity. Although this dataset is not the best one to assess precision, the results do not support the authors' claim: "Taken together, these results suggest JAFFAL is highly accurate".

*We have remade Figure 3C to demonstrate the performance of JAFFAL on MCF-7 better. We now show the number of previously validated fusions against the number of other fusions. We feel this is more consistent with other subfigures in Figure 3 and more precise language. Not all previously identified fusions will be present (expressed) in our samples due to differences between cell line batches, therefore the recall can not be directly assessed on the cell lines data. Instead JAFFAL’s sensitivity is shown in our fusion simulation. Similarly, not all “other reported” fusions are false positives and therefore precision is difficult to assess.*

*Figure 3C now also only includes high confidence fusion calls, as this is the classification we recommended for fusion detection and therefore the correct one to measure our pipeline's accuracy on.*

The authors also claim "We show that fusions can be accurately detected in long read data with JAFFAL, providing better accuracy than other long read fusion finders". This sentence should say "than another long read fusion finder", as the comparison is only made with LongGF. Additionally, given the high rate of potential false positives or the lack of a strategy to confidently separate true and separate positives, it is not clear that fusion detection with long-reads is "accurate".

*We have reworded this sentence to “We show that fusions can be accurately detected in long read data with JAFFAL, with similar performance as state-of-the-art methods applied to short read data.” Throughout the manuscript we have demonstrated using simulation and real data that our high confidence ranking is excellent at separating true and false positives.*

The authors claim "We show JAFFAL is the most accurate fusion finder available for noisy long read data, allowing fusions to be detected in long read data with similar accuracy as short reads." This should be toned down, as it is the best of two, and in the comparisons shown in the current version of the manuscript. In the previous version, in the simulated ONT data without background, LongGF faired quite well.

*This sentence has been reworded in the revised manuscript to “We show JAFFAL is an*

*accurate fusion finder available for noisy long read data, allowing fusions to be detected in long read data with similar accuracy as short reads.”*

Perhaps the manuscript is too focused on presenting a novel and accurate method. However, the results do not fully support the authors' claims. On the other hand, the manuscript is possibly presenting the most comprehensive study to date on gene-fusions from long read data. This could provide an opportunity to rewrite the article as a more realistic perspective of the limitations and opportunities of long read sequencing to study gene fusions.

*The manuscript is indeed focused on presenting a novel and accurate method, and we plan to resubmit the revised manuscript as a methods paper.*

**Third round of review**

**Reviewer 1**

The authors have added additional analyses about the false positives. And this is very much welcome. However, how they have presented this information (Supplementary Figure 2) makes it hard to compare it with the rest of the analyses. The Supp. Fig. 2 plots the number of false positives per Gbp sequenced, whereas Table 1 and Table 2 shows the results relative to the total number of reads, which ranges between ~25M and 44M reads for ONT. This makes it very hard to understand the number of false positives expected with this number of reads.

It is still not possible to clearly know what the expected number of false positives would be for datasets of a size comparable to those shown in Tables 1 and 2. The authors should plot for example the number of false positives per 1M reads, to make it easier to interpret and comparable to the other data presented.

The authors should also acknowledge in the conclusions that to recover known fusions a large number of reads is required. For instance, for the cancer cell lines (Table 2), they are using >30M reads, and in some cases, not all known fusions are recovered. This is critical. Readers should know what level of sequencing depth is required to potentially recover fusions in new samples. As the conclusion reads now, fusions would appear to be retrieved from any PacBio/ONT experiment. But that's not entirely true, as the datasets must be particularly large.

**Authors Response**

**Point-by-point responses to the reviewers’ comments:**

*We have updated Supplementary Figure 2. To show false positives per million reads, as suggested by the reviewer.*

*Finally, we have added the following sentence to the conclusion to address the final point from the reviewer:*

*“We validated JAFFAL on datasets with up to 44 million reads and even for deeply sequenced samples, some fusions may be missed due to low expression.”*
